# Correction: Active and passive mechanics for rugose terrain traversal in centipedes

**DOI:** 10.1242/jeb.247054

**Published:** 2024-02-13

**Authors:** Kelimar Diaz, Eva Erickson, Baxi Chong, Daniel Soto, Daniel I. Goldman

There was an error in *J. Exp. Biol.* (2023) 226, jeb244688 (doi:10.1242/jeb.244688).

**Fig. 1C,D (corrected panels).**
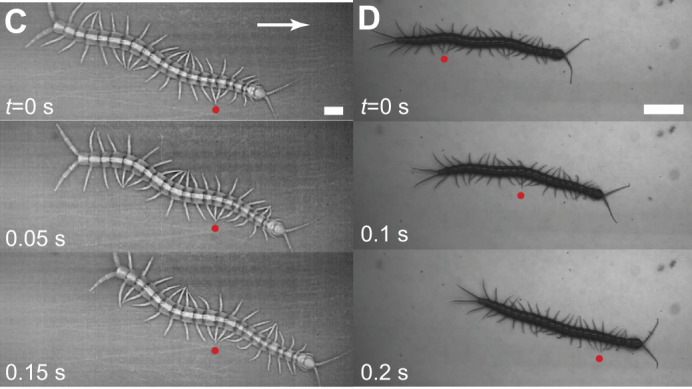
**Centipedes with distinct limb-stepping patterns.** Image sequence showing (C) *S. polymorpha* and (D) *S. sexspinosus* running on foam core. Note that for *S. polymorpha*, snapshots go to 0.15 s, not 0.2 s.

**Fig. 1C,D (original panels).**
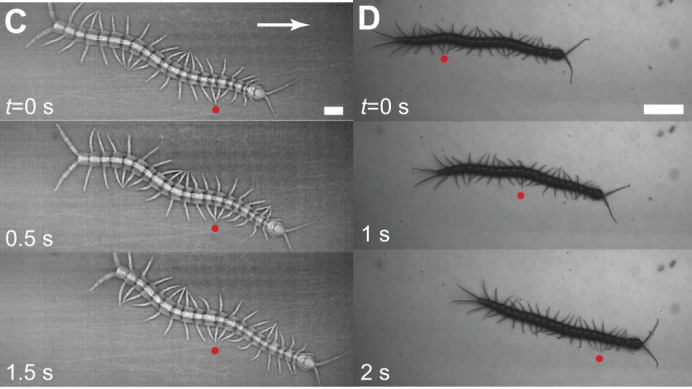
**Centipedes with distinct limb-stepping patterns.** Image sequence showing (C) *S. polymorpha* and (D) *S. sexspinosus* running on foam core. Note that for *S. polymorpha*, snapshots go to 1.5 s, not 2 s.

The timestamps in Fig. 1C,D are incorrect by a factor of 10. The corrected and original versions of Fig. 1C,D are shown below. Both the online full text and PDF versions of the paper have been corrected. The authors apologise to the readers for this error, which does not impact the results or conclusions of the paper.

